# Sudden cardiac death after myocardial infarction: individual participant data from pooled cohorts

**DOI:** 10.1093/eurheartj/ehae326

**Published:** 2024-10-08

**Authors:** Niels Peek, Gerhard Hindricks, Artur Akbarov, Jan G P Tijssen, David A Jenkins, Zoher Kapacee, Le Mai Parkes, Rob J van der Geest, Enrico Longato, Daniel Sprague, Youssef Taleb, Marcus Ong, Christopher A Miller, Alireza Sepehri Shamloo, Christine Albert, Petra Barthel, Serge Boveda, Frieder Braunschweig, Jens Brock Johansen, Nancy Cook, Christian de Chillou, Petra Elders, Jonas Faxén, Tim Friede, Laura Fusini, Chris P Gale, Jiri Jarkovsky, Xavier Jouven, Juhani Junttila, Josef Kautzner, Antti Kiviniemi, Valentina Kutyifa, Christophe Leclercq, Daniel C Lee, Jill Leigh, Radosław Lenarczyk, Francisco Leyva, Michael Maeng, Andrea Manca, Eloi Marijon, Ursula Marschall, Jose Luis Merino, Lluis Mont, Jens Cosedis Nielsen, Thomas Olsen, Julie Pester, Gianluca Pontone, Ivo Roca, Georg Schmidt, Peter J Schwartz, Christian Sticherling, Mahmoud Suleiman, Milos Taborsky, Hanno L Tan, Jacob Tfelt-Hansen, Holger Thiele, Gordon F Tomaselli, Tom Verstraelen, Manickavasagar Vinayagamoorthy, Kevin Kris Warnakula Olesen, Arthur Wilde, Rik Willems, Katherine C Wu, Markus Zabel, Glen P Martin, Nikolaos Dagres

**Affiliations:** Division of Informatics, Imaging and Data Science, Faculty of Biology, Medicine and Health, University of Manchester, Manchester, UK; The Healthcare Improvement Studies Institute (THIS Institute), Department of Public Health and Primary Care, University of Cambridge, Cambridge, UK; Department of Cardiology, Angiology and Intensive Care Medicine, Deutsches Herzzentrum der Charité, Campus Charité Mitte, Charitéplatz 1, 10117 Berlin, Germany; Department of Electrophysiology, Heart Center Leipzig, Strumpellstr. 39, 04289 Leipzig, Germany; Division of Informatics, Imaging and Data Science, Faculty of Biology, Medicine and Health, University of Manchester, Manchester, UK; Clinical Epidemiology and Biostatistics, The AMC, Amsterdam, The Netherlands; Division of Informatics, Imaging and Data Science, Faculty of Biology, Medicine and Health, University of Manchester, Manchester, UK; Division of Informatics, Imaging and Data Science, Faculty of Biology, Medicine and Health, University of Manchester, Manchester, UK; Division of Informatics, Imaging and Data Science, Faculty of Biology, Medicine and Health, University of Manchester, Manchester, UK; Department of Radiology, Leiden University Medical Center, Leiden, The Netherlands; Department of Information Engineering, University of Padova, Padova, Italy; Spectra Analytics, London, UK; Spectra Analytics, London, UK; Spectra Analytics, London, UK; Division of Cardiovascular Sciences, School of Medical Sciences, Faculty of Biology, Medicine and Health, University of Manchester, Manchester Academic Health Science Centre, Manchester, UK; Department of Cardiology, Angiology and Intensive Care Medicine, Deutsches Herzzentrum der Charité, Campus Charité Mitte, Charitéplatz 1, 10117 Berlin, Germany; Department of Electrophysiology, Heart Center Leipzig, Strumpellstr. 39, 04289 Leipzig, Germany; Department of Cardiology, Smidt Heart Institute, Cedars Sinai Medical Center, Los Angeles, CA, USA; Klinikum rechts der Isar, Technische Universität München, Munich, Germany; Cardiology—Heart Rhythm Management Department, Clinique Pasteur, Toulouse, France; Department of Cardiology, Karolinska University Hospital, Stockholm, Sweden; Department of Cardiology, Department of Cardiology Odense, Odense University Hospital, Syddanmark, Denmark; Brigham and Women’s Hospital, Harvard Medical School, Boston, MA, USA; Département de Cardiologie, CHRU de Nancy, Nancy, France; Department of General Practice and Elderly Care, Medicine, Vrije Universiteit Amsterdam, Amsterdam, The Netherlands; Department of Physiology and Pharmacology, Karolinska Institutet, Stockholm, Sweden; Department of Medical Statistics, University Medical Center Göttingen, Göttingen, Germany; Department of Cardiovascular Imaging, Centro Cardiologico Monzino IRCCS, Milan, Italy; Leeds Institute of Cardiovascular and Metabolic Medicine, University of Leeds, Leeds, UK; Institute of Biostatistics and Analyses, Faculty of Medicine, Masaryk University, Brno, Czech Republic; Medical and Surgical Department of Cardiology, Georges Pompidou European Hospital, Paris, France; Research Unit of Internal Medicine, Medical Research Center Oulu, University of Oulu and Oulu University Hospital, Oulu, Finland; Institute of Clinical and Experimental Medicine, University Hospital Olomouc, Moravia, Czech Republic; Research Unit of Internal Medicine, Medical Research Center Oulu, University of Oulu and Oulu University Hospital, Oulu, Finland; University of Rochester Medical Center, Clinical Cardiovascular Research Center, Rochester, NY, USA; Service de Cardiologie et Maladies Vasculaires, CHU Pontchaillou, Rennes, France; School of Medicine, Northwestern University Feinberg, Chicago, USA; Boston Scientific Corporation, St. Paul, MN, USA; Division of Medical Sciences in Zabrze, Department of Cardiology, Congenital Heart Diseases and Electrotherapy, Silesian Center of Heart Diseases, The Medical University of Silesia, Katowice, Poland; Aston Medical School, Aston University, Aston Triangle, Birmingham, UK; Department of Cardiology, Aarhus University Hospital, Aarhus, Denmark; Centre for Health Economics, University of York, York, UK; Division of Cardiology, European Georges Pompidou Hospital, Paris, France; Barmer, Germany; Arrhythmia and Robotic Electrophysiology Unit, La Paz University Hospital, Madrid, Spain; Hospital Clinic, University of Barcelona, Catalonia, Spain; Department of Cardiology, Aarhus University Hospital, Aarhus, Denmark; Department of Clinical Medicine, Aarhus University, Aarhus, Denmark; Department of Cardiology, Department of Cardiology Odense, Odense University Hospital, Syddanmark, Denmark; Brigham and Women’s Hospital, Harvard Medical School, Boston, MA, USA; Department of Cardiovascular Imaging, Centro Cardiologico Monzino IRCCS, Milan, Italy; Hospital Clinic, University of Barcelona, Catalonia, Spain; Klinikum rechts der Isar, Technische Universität München, Munich, Germany; Center for Cardiac Arrhythmias of Genetic Origin, IRCCS Istituto Auxologico Italiano, Milan, Italy; Department of Cardiology, University Hospital Basel, University Basel, Basel, Switzerland; Department of Cardiology, Rambam Health Care Campus, Haifa, Israel; Department of Internal Medicine I—Cardiology, Olomouc University Hospital, Moravia, Czech Republic; Department of Clinical and Experimental Cardiology, Amsterdam University Medical Center AMC, University of Amsterdam, Amsterdam, Netherlands; Department of Cardiology, The Heart Centre, Copenhagen University Hospital, Rigshospitalet, Copenhagen, Denmark; Heart Center Leipzig at the University of Leipzig, Leipzig, Germany; Albert Einstein College of Medicine, Bronx, NY; Department of Cardiology, Amsterdam UMC location University of Amsterdam, Amsterdam, The Netherlands; Brigham and Women’s Hospital, Harvard Medical School, Boston, MA, USA; Department of Cardiology, Aarhus University Hospital, Aarhus, Denmark; Department of Cardiology, Amsterdam UMC location University of Amsterdam, Amsterdam, The Netherlands; Department of Cardiovascular Sciences, University of Leuven, Leuven, Belgium; Cardiology, University Hospitals Leuven, Leuven, Belgium; Division of Cardiology, Johns Hopkins University School of Medicine, Baltimore, MD, USA; Department of Cardiology and Pneumology, Heart Center, University Medical Center Goettingen, Göttingen, Germany; Division of Informatics, Imaging and Data Science, Faculty of Biology, Medicine and Health, University of Manchester, Manchester, UK; Department of Cardiology, Angiology and Intensive Care Medicine, Deutsches Herzzentrum der Charité, Campus Charité Mitte, Charitéplatz 1, 10117 Berlin, Germany; Department of Electrophysiology, Heart Center Leipzig, Strumpellstr. 39, 04289 Leipzig, Germany

**Keywords:** Implantable cardioverter-defibrillator, Myocardial infarction, Primary prevention, Sudden cardiac death

## Abstract

**Background and Aims:**

Risk stratification of sudden cardiac death after myocardial infarction and prevention by defibrillator rely on left ventricular ejection fraction (LVEF). Improved risk stratification across the whole LVEF range is required for decision-making on defibrillator implantation.

**Methods:**

The analysis pooled 20 data sets with 140 204 post-myocardial infarction patients containing information on demographics, medical history, clinical characteristics, biomarkers, electrocardiography, echocardiography, and cardiac magnetic resonance imaging. Separate analyses were performed in patients (i) carrying a primary prevention cardioverter-defibrillator with LVEF ≤ 35% [implantable cardioverter-defibrillator (ICD) patients], (ii) without cardioverter-defibrillator with LVEF ≤ 35% (non-ICD patients ≤ 35%), and (iii) without cardioverter-defibrillator with LVEF > 35% (non-ICD patients >35%). Primary outcome was sudden cardiac death or, in defibrillator carriers, appropriate defibrillator therapy. Using a competing risk framework and systematic internal–external cross-validation, a model using LVEF only, a multivariable flexible parametric survival model, and a multivariable random forest survival model were developed and externally validated. Predictive performance was assessed by random effect meta-analysis.

**Results:**

There were 1326 primary outcomes in 7543 ICD patients, 1193 in 25 058 non-ICD patients ≤35%, and 1567 in 107 603 non-ICD patients >35% during mean follow-up of 30.0, 46.5, and 57.6 months, respectively. In these three subgroups, LVEF poorly predicted sudden cardiac death (*c*-statistics between 0.50 and 0.56). Considering additional parameters did not improve calibration and discrimination, and model generalizability was poor.

**Conclusions:**

More accurate risk stratification for sudden cardiac death and identification of low-risk individuals with severely reduced LVEF or of high-risk individuals with preserved LVEF was not feasible, neither using LVEF nor using other predictors.

## Structured Graphical Abstract

**Structured Graphical Abstract:**
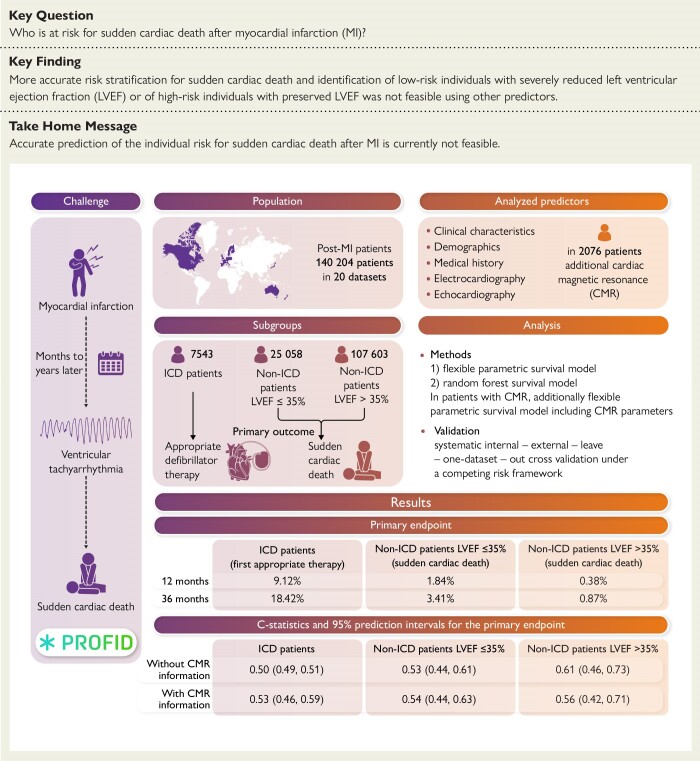
Brief summary of the background, methodology, and results of the PROFID data analysis. ICD patients, patients with left ventricular ejection fraction ≤ 35% who had received a cardioverter-defibrillator implantation for primary prevention of sudden cardiac death; non-ICD patients ≤35%, patients who did not carry a cardioverter-defibrillator and had a left ventricular ejection fraction ≤ 35%; and non-ICD patients >35%, patients who did not carry a cardioverter-defibrillator and had a left ventricular ejection fraction > 35%. Detailed inclusion and exclusion criteria are provided in the text. CMR, cardiac magnetic resonance; ICD, implantable cardioverter-defibrillator; LVEF, left ventricular ejection fraction; MI, myocardial infarction.

**See the editorial comment for this article ‘Refining the stratification of sudden cardiac death risk after myocardial infarction—beyond ejection fraction’, by E.C. Ajufo and U.B. Tedrow, https://doi.org10.1093/eurheartj/ehae272.**

## Introduction

Sudden cardiac death is the leading cause of death, accounting for ≈20% of deaths.^1,2^ Patients with previous myocardial infarction are at particular risk due to life-threatening ventricular arrhythmias.^3^ The implantable cardioverter-defibrillator detects and terminates these arrhythmias. However, defibrillator therapy is limited by the profound difficulty to identify patients at elevated sudden cardiac death risk as candidates for implantation.

Historic trials, which is restricted by design inclusion to patients with reduced left ventricular ejection fraction (LVEF), found that cardioverter-defibrillator implantation improves survival in patients with severely impaired LVEF,^4,5^ a non-specific risk factor for both sudden and non-sudden cardiac death.^6^ Current guidelines therefore recommend prophylactic cardioverter-defibrillator implantation in these patients.^7,8^ This strategy has significant shortcomings.^9^ Treatment advances, in particular guideline-directed medical therapy, have led to substantial reduction of the sudden cardiac death risk in patients with reduced LVEF,^10^ and most of currently implanted cardioverter-defibrillators are not required during their life cycle.^11^ The identification of the many low-risk patients not requiring defibrillator protection is crucial to avoid unnecessary implantations. Additionally, many sudden cardiac deaths occur in patients with mildly reduced or preserved left ventricular function.^1,2^ In this population, risk stratification attempts remain scarce.

The aim of this analysis was to investigate whether use of LVEF and of a broad spectrum of further candidate predictors allows identification of low-risk patients with severely reduced LVEF not needing defibrillator protection and of high-risk patients with mildly reduced or preserved LVEF as candidates for targeted defibrillator implantation. The presented work is part of the PROFID project.

## Methods

The structure and reporting follow the Transparent Reporting of a multivariable prediction model for Individual Prognosis Or Diagnosis (TRIPOD) statement.^12^

### Data sources and study population

Twenty data sets from Europe, the USA, and Israel were analysed including (i) cohort data sets of patients who had coronary artery disease with previous myocardial infarction and/or ischaemic cardiomyopathy with reduced LVEF (<50%) and were entered into the data set at some time after the infarction; (ii) cohort data sets of patients who had acute myocardial infarction and were entered into the data set at the time of the acute event; (iii) cohort data sets of patients who underwent prophylactic cardioverter-defibrillator implantation for primary prevention of sudden cardiac death after previous myocardial infarction and were entered into the data set at the time of device implantation, and (iv) data sets from randomized controlled trials, which compared different cardioverter-defibrillator programming settings or the outcome of patients receiving a cardioverter-defibrillator against medical treatment in patients with prior myocardial infarction and/or ischaemic cardiomyopathy with severely reduced LVEF. Details are given in Supplementary data online, *Materials*.

### Participant inclusion and exclusion criteria

Included in the analysis were patients older than 18 years with either (i) previous ST-elevation and/or non-ST-elevation myocardial infarction regardless of LVEF or (ii) coronary artery disease with ischaemic cardiomyopathy and reduced LVEF (<50%).^8^ The following patients were excluded: (i) patients carrying at baseline a cardioverter-defibrillator for secondary sudden cardiac death prevention; (ii) patients who had received a cardioverter-defibrillator within 40 days after infarction; (iii) patients with a cardiac resynchronization therapy device at baseline; (iv) patients with coexisting non-ischaemic cardiomyopathy (such as dilated, hypertrophic, or restrictive cardiomyopathy), coexisting primary electrical arrhythmic disease (such as long QT or Brugada syndrome), or coexisting congenital heart disease; and (v) patients who died or experienced the outcome within the first 40 days after the index infarction.

To account for the use of a surrogate endpoint in patients carrying a defibrillator, we analysed separately (i) patients with LVEF ≤ 35% who had received a cardioverter-defibrillator implantation for primary prevention of sudden cardiac death, hitherto called ‘ICD patients’, and (ii) patients who did not carry a cardioverter-defibrillator within 3 months after the index myocardial infarction event, hitherto called ‘non-ICD patients’. In the latter, separate analyses were performed in (iia) patients with LVEF ≤ 35%, called ‘non-ICD patients ≤35%’, and (iib) patients with LVEF > 35% called ‘non-ICD patients >35%’.

### Primary outcome

In non-ICD patients, the primary outcome was sudden cardiac death/sudden cardiac arrest. The definition of sudden cardiac death that was applied in each data set is given in the ‘Description of data sets’ in the Supplementary data online, *Material*. In most cases, the Hinkle–Thaler definition was applied.^13^ In two data sets with non-ICD patients, the primary outcome included additionally life-threatening ventricular arrhythmias (ventricular fibrillation or ventricular tachycardia). We modelled time-to-the-primary outcome accounting for the competing risk of death from other causes through the Fine and Gray competing risk framework.^14^ Time zero was defined as 40 days after the index infarction or at the start of study enrolment, whichever occurred latest.

In ICD patients, the primary outcome, as best available surrogate for sudden cardiac death in this population, was defined as appropriate therapy (anti-tachycardia pacing or shock) delivered by the defibrillator or, if anti-tachycardia pacing data were not collected, appropriate shock. Time-to-first-appropriate therapy/shock was modelled accounting for the competing risk of death from other cause (prior to first appropriate shock/therapy) through the Fine and Gray competing risk framework.^14^ Time zero was defined as the point of defibrillator implantation.

Some non-ICD patients underwent defibrillator implantation during follow-up. If implantation took place for secondary prevention of sudden cardiac death, such events were considered to constitute the primary outcome. If implantation occurred for primary prevention, this was not considered an outcome; patients were continued to be modelled until they either died suddenly, died from other causes, or were censored (intention-to-treat analysis).

### Candidate predictors

We used individual participant data pertaining to demographics, medical history, clinical parameters, biomarkers, medication, electrocardiography, and echocardiography (see Supplementary data online, *Table S1*) and, in a subset of data sets and patients, cardiac magnetic resonance imaging. To address the expected heterogeneity in prevalence of the primary outcome across data sets, a categorical ‘risk geography’ predictor variable based on the cardiovascular disease risk regions of the World Health Organization was added (see Supplementary data online, *Figures S1* and *S2*).

### Missing data

Only those predictors were considered that were present in ≥75% of observations and recorded in the majority of data sets (see Supplementary data online, *Table S2*). Missing data in predictor variables (both systematically missing across entire data sets and sporadically missing within certain data sets) were imputed using fuzzy K-means.^15^ As sensitivity, an ‘uncapped analysis’ was performed considering all available candidate predictors (all variables listed in Supplementary data online, *Table S1*; results were quantitatively similar and are available upon request).

### Statistical analysis

Continuous variables are reported as mean (standard deviation) or median (interquartile range) as appropriate and categorical variables as frequencies of occurrence (percentage). Time-to-event was visually explored by cumulative incidence plots of the outcome or death from other causes.

To account for the fact that cardiac magnetic resonance imaging was available only in a patient subset, the analysis consisted of two phases. In Phase 1, all candidate predictors were considered except parameters derived from cardiac magnetic resonance imaging. In Phase 2, the analysis was updated with inclusion of candidate predictors from cardiac magnetic resonance imaging.

In Phase 1, we first assessed the predictive performance of LVEF for sudden cardiac death. Using systematic internal–external leave-one-data set-out cross-validation,^16,17^ a flexible parametric survival model was fit under a Fine and Gray competing risk framework with LVEF as sole, continuous predictor variable. In brief, leave-one-data set-out cross-validation means that each time one data set was left out, a model with LVEF as sole predictor was built in all remaining data sets and the model was then validated in the data set that had been left out. This cycle was repeated for every data set. The resulting estimates of predictive performance of LVEF for sudden cardiac death, one per data set, were then combined by random effects meta-analysis providing the overall estimate of the predictive performance of LVEF across all data sets as well as the associated prediction interval, which gives the expected performance in a new data set that is similar to the analysed ones.^18^ This was done in each of the three patient subgroups separately.

To assess whether consideration of further candidate predictors apart from LVEF improved the prediction of sudden cardiac death, we developed and externally validated (again using systematic internal–external leave-one-data set-out cross-validation) multivariable prediction models for sudden cardiac death considering two different analytical methods within a competing risk framework: (i) flexible parametric survival model^19,20^ and (ii) random forest survival model^21^ applying again the process described above. To select the candidate predictors for the flexible parametric survival models, backwards selection under Bayesian information criteria stopping rule was applied.

In Phase 2, we assessed whether parameters from cardiac magnetic resonance imaging improved prediction of sudden cardiac death over LVEF alone. Seven data sets included information from cardiac magnetic resonance imaging and were included in the Phase 2 analysis: six of the data sets from Phase 1 analysis and an additional data set that was not included in Phase 1 as it contained mainly information related to cardiac magnetic resonance imaging. Within these data sets, two models were fit: (i) a flexible parametric survival model with age, sex, and LVEF as only covariates and (ii) a flexible parametric survival model with age, sex, and LVEF, plus core scar and greyzone. For definition of core scar and greyzone, the application of the full-width-half-maximum, 2 SD, 3 SD, and 5 SD methods was considered as previously described.^22^ The definition resulting in the best predictive performance across all data sets was selected. As with Phase 1, systematic leave-one-data set-out cross-validation was performed to validate the models. Given the smaller number of data sets and events, results were pooled using fixed effect meta-analysis.

The predictive performance of all models was assessed using discrimination and calibration within the competing risk framework at a prediction horizon of 36 months post time zero. Discrimination was assessed using time-dependent *c*-statistics with ideal value of 1, whereas 0.5 indicates random performance. Calibration was assessed by comparing the observed cumulative incidence function for the primary endpoint with the cumulative incidence function predicted by the model at 36 months, hereto called ‘observed:expected ratio’, with ideal value of 1. To visualize discrimination performance, within each data set of each study population, patients were split into three equally sized groups based on the 36-month risk for each model separately. A cumulative incidence plot was produced in each of the three study populations, stratified by the three risk groups.

Analyses were undertaken in R version 3.6.1,^23^ Stata (version. 16.1; StataCorp), and Python.

## Results

### Phase 1 analysis

In Phase 1, i.e. the analysis excluding magnetic resonance imaging information, 140 204 patients across all data sets fulfilled the inclusion and exclusion criteria, comprised of 7543 ICD patients, 25 058 non-ICD patients ≤35%, and 107 603 non-ICD patients >35%. Mean age was 63.8, 72.6, and 68.3 years, respectively. Baseline characteristics are shown in *Table 1*. The whole range of LVEF was represented. There were 1326 primary outcomes in ICD patients, 1193 non-ICD patients ≤35% and 1567 non-ICD patients >35% during mean follow-up of 30.0, 46.5, and 57.6 months. The cumulative incidence function of the primary outcome is given in *Table 2* and Supplementary data online, *Figure S3*.

**Table 1:** Baseline characteristics across the three subgroups in the Phase 1 analysis, i.e. the analysis excluding cardiac magnetic resonance imaging parameters, and in the Phase 2 analysis, i.e. the analysis including cardiac magnetic resonance imaging parameters

Phase 1 analysis (excluding cardiac magnetic resonance imaging parameters)				
Variable	ICD patients (*n* = 7543)	Non-ICD patients ≤35% (*n* = 25 058)	Non-ICD patients >35% (*n* = 107 603)	Missing data *n* (%)
Demographics				
Age (years)	63.8 (10.7)	72.6 (11.7)	68.3 (11.9)	2 (0%)
Sex (male)	6410 (85.0%)	17 145 (68.4%)	72 813 (67.7%)	1 (0%)
Medical history				
Prior percutaneous coronary intervention	1571 (20.8%)	3576 (14.3%)	16 048 (14.9%)	10 140 (7.2%)
Prior coronary bypass graft surgery	1528 (20.3%)	2870 (11.5%)	7428 (6.9%)	8401 (6.0%)
Smoking	2113 (28.0%)	13 740 (54.8%)	60 681 (56.4%)	11 230 (8.0%)
Clinical characteristics				
Body mass index, kg/m^2^	28.2 (5.4)	26.6 (4.6)	27.3 (4.6)	18 583 (13.3%)
Hypertension	2402 (31.8%)	12 528 (50.0%)	53 561 (49.8%)	5586 (4.0%)
Diabetes	2049 (27.2%)	6847 (27.3%)	21 848 (20.3%)	1958 (1.4%)
Myocardial infarction type				11 903 (8.49%)
ST-segment elevation	Not available^a^	8293 (33.1%)	28 013 (26.0%)	
Non-ST-segment elevation	Not available^a^	14 312 (57.1%)	70 140 (65.2%)	
Atrial fibrillation or atrial flutter	1273 (16.9%)	5379 (21.5%)	11 473 (10.7%)	9723 (6.9%)
Estimated glomerular filtration rate (mL/min/1.73 m^2^), median (interquartile range)	69.0 (51.4–85.0)	68.0 (49.9–85.0)	79.6 (62.6–91.8)	9020 (6.4%)
Electrocardiography				
Left bundle branch block	391 (5.2%)	3100 (12.4%)	3177 (3.0%)	11 654 (8.3%)
Medication				
Angiotensin-converting enzyme inhibitors and/or angiotensin receptor blockers	5029 (66.7%)	21 527 (85.9%)	82 760 (76.9%)	2259 (1.6%)
Beta-blockers	5249 (69.6%)	22 381 (89.3%)	95 197 (88.5%)	1581 (1.1%)
Diuretics	4276 (56.7%)	13 086 (52.2%)	23 988 (22.3%)	1828 (1.3%)
Antiplatelet drugs	4266 (56.6%)	22 642 (90.4%)	99 610 (92.6%)	6981 (5.0%)
Oral anticoagulants	1582 (21.0%)	3977 (15.9%)	7444 (6.9%)	12 711 (9.0%)
Lipid-lowering medication	3163 (41.9%)	20 669 (82.5%)	98 954 (92.0%)	3440 (2.5%)
Echocardiography				
Left ventricular ejection fraction (%)	26.5 (5.9)	29.9 (6.3)	55.2 (7.1)	94 (0.1%)

Phase 2 analysis (including cardiac magnetic resonance imaging parameters)				
Variable	ICD patients (*n* = 514)	Non-ICD patients ≤35% (*n* = 576)	Non-ICD patients >35% (*n* = 986)	Missing data *n* (%)
Sex (male)	381 (74.1%)	466 (80.9%)	771 (78.2%)	0
Age (years)	63.0 (11.3)	65.9 (11.5)	64.1 (11.8)	0
Left ventricular ejection fraction (%)	25.2 (6.7)	25.9 (6.3)	48.9 (10.8)	0
Extent of core scar (g) median (interquartile range)	24.5 (9.3–44.8)	30.5 (11.6–49.8)	18.6 (3.2–35.4)	0
Extent of greyzone (g) median (interquartile range)	12.7 (4.9–20.7)	15.0 (9.4–21.1)	9.8 (4.2–14.8)	0

Unless stated otherwise, values give mean numbers with standard deviation in parentheses or absolute numbers with percentages in parentheses as appropriate. Characteristics shown are for primary data, i.e. prior to imputation of missing values. ICD patients, patients with left ventricular ejection fraction ≤ 35% who had received a cardioverter-defibrillator implantation for primary prevention of sudden cardiac death. Non-ICD patients ≤35%, patients who did not carry a cardioverter-defibrillator and had a left ventricular ejection fraction ≤ 35%. Non-ICD patients >35%, patients who did not carry a cardioverter-defibrillator and had a left ventricular ejection fraction > 35%. The column ‘Missing data’ gives numbers of patients with missing data across all three subgroups.

^a^ICD patients were entered into the data sets at some time after the myocardial infarction event and the information on ST- or non-ST-segment elevation infarction was not available. Core scar was defined by the 5 SD method, and greyzone was defined by the 3 SD minus 5 SD method.

**Table 2:** Fine and Gray cumulative incidence of the primary endpoint in the three patient subgroups at 12 and 36 months in the Phase 1 analysis, i.e. the analysis excluding cardiac magnetic resonance imaging parameters, and in the Phase 2 analysis, i.e. the analysis including cardiac magnetic resonance imaging parameters

Time point (months after time zero)	ICD patients incidence (95% confidence interval) of the endpoint, first appropriate therapy	Non-ICD patients ≤35% incidence (95% confidence interval) of the endpoint, sudden cardiac death^a^	Non-ICD patients >35% incidence (95% confidence interval) of the endpoint, sudden cardiac death^a^
Phase 1 analysis (excluding cardiac magnetic resonance imaging parameters)			
12	9.12% (8.48%, 9.77%)	1.84% (1.68%, 2.01%)	0.38% (0.34%, 0.41%)
36	18.42% (17.44%, 19.39%)	3.41% (3.18%, 3.63%)	0.87% (0.81%, 0.92%)
Phase 2 analysis (including cardiac magnetic resonance imaging parameters)			
12	7.49% (5.19%, 9.78%)	3.66% (2.12%, 5.19%)	0.41% (0.01%, 0.81%)
36	17.43% (14.05%, 20.82%)	7.34% (5.14%, 9.54%)	1.89% (1.02%, 2.75%)

ICD patients, patients with left ventricular ejection fraction ≤ 35% who had received a cardioverter-defibrillator implantation for primary prevention of sudden cardiac death. Non-ICD patients ≤35%, patients who did not carry a cardioverter-defibrillator and had a left ventricular ejection fraction ≤ 35%. Non-ICD patients >35%, patients who did not carry a cardioverter-defibrillator and had a left ventricular ejection fraction > 35%.

^a^In two data sets, the primary outcome included additionally life-threatening ventricular arrhythmias (ventricular fibrillation or ventricular tachycardia).

**Figure 1:**
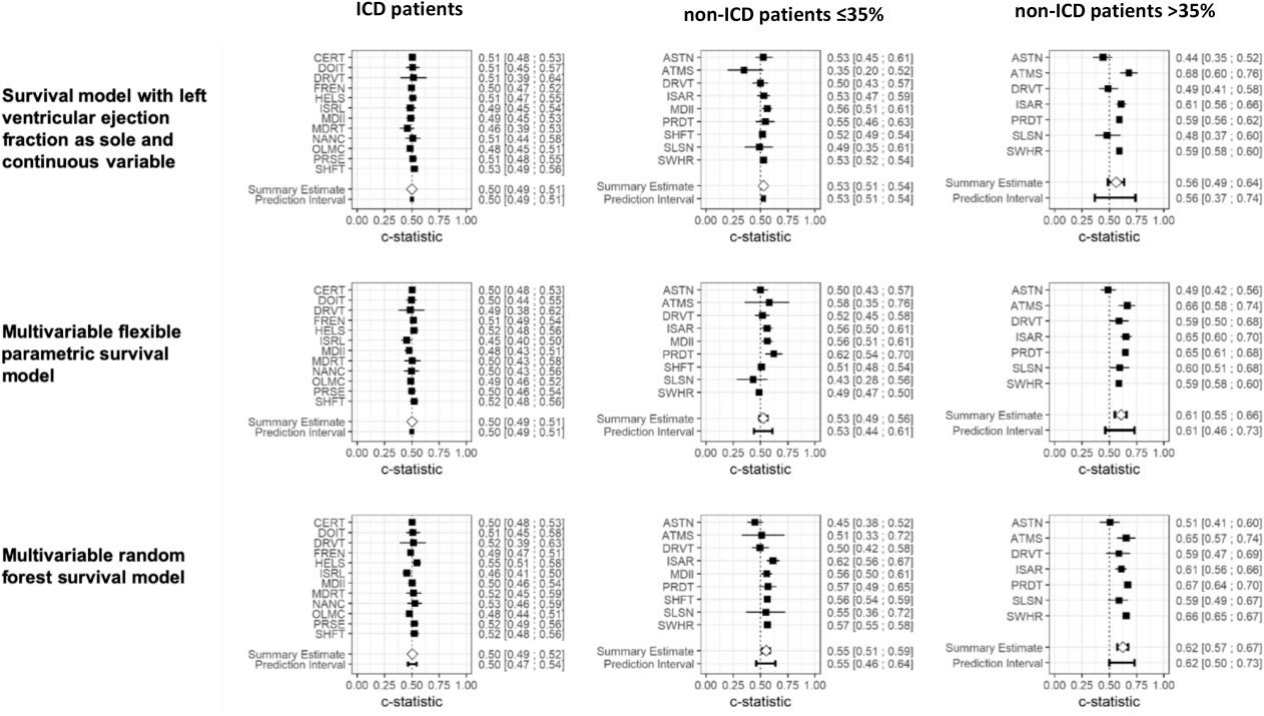
Forest plot of the *c*-statistic for the prediction of risk for the primary endpoint at 36 months across the three subgroups in the Phase 1 analysis, i.e. the analysis excluding cardiac magnetic resonance imaging parameters. ICD patients, patients with left ventricular ejection fraction ≤ 35% who had received a cardioverter-defibrillator implantation for primary prevention of sudden cardiac death; non-ICD patients ≤35%: patients who did not carry a cardioverter-defibrillator and had a left ventricular ejection fraction ≤ 35%; and non-ICD patients >35%, patients who did not carry a cardioverter-defibrillator and had a left ventricular ejection fraction > 35%. In ICD patients, endpoint was first appropriate therapy, and in non-ICD patients ≤35% and non-ICD patients >35%, endpoint was sudden cardiac death. In two data sets with non-ICD patients, the primary endpoint included additionally life-threatening ventricular arrhythmias (ventricular fibrillation or ventricular tachycardia). Please note that the leave-one-data set-out cross-validation was applied meaning that each time one data set was left out, a model was built in all remaining data sets and the model was then applied in the data set that had been left out. This cycle was then repeated for every data set. The resulted estimates of predictive performance for the primary endpoint, one per data set, were then combined by random effects meta-analysis providing the overall estimate of the predictive performance of ejection fraction across all data sets as well as the associated prediction interval, which gives the expected performance in a new data set that is similar to the analysed ones. A wide prediction interval indicates limited generalizability to a new data set. To select the candidate predictors for the multivariable models, only those predictors were considered that were present in ≥75% of observations and recorded in the majority of data sets. For the multivariable flexible parametric survival models, backwards selection under Bayesian information criteria stopping rule was applied. The named data sets on the y-axis denote the data set left-out for model development and then used to validate the subsequent model to produce the corresponding performance estimates shown. For abbreviations of the individual data sets, please see the ‘Description of data sets’ in the Supplementary data online, *Material*

**Figure 2:**
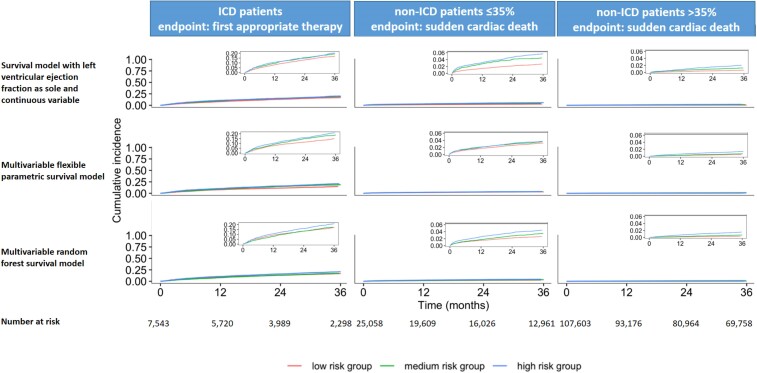
Fine and Gray cumulative incidence of the primary endpoint stratified by predicted risk categories obtained from each model for the three subgroups in the Phase 1 analysis. The figure depicts Fine and Gray cumulative incidence plots of the primary endpoint stratified by predicted risk, for the three cohorts in the Phase 1 analysis. In each iteration of the leave-one-data set cross-validation loop, patients in the validation data set were split into three equally sized groups (low, medium, and high) based on 36-month risk, predicted by the model that was developed in the remaining data sets. After completing the leave-one-data set cross-validation process, all patients in the low-risk group were pooled to produce the cumulative incidence plot, and the same happened for the medium-risk and high-risk groups. ICD patients, patients with left ventricular ejection fraction ≤ 35% who had received a cardioverter-defibrillator implantation for primary prevention of sudden cardiac death; non-ICD patients ≤35%, patients who did not carry a cardioverter-defibrillator and had a left ventricular ejection fraction ≤ 35%; and non-ICD patients >35%, patients who did not carry a cardioverter-defibrillator and had a left ventricular ejection fraction > 35%. In ICD patients, endpoint was first appropriate therapy, and in non-ICD patients ≤35% and non-ICD patients >35%, endpoint was sudden cardiac death. In two data sets with non-ICD patients, the primary endpoint included additionally life-threatening ventricular arrhythmias (ventricular fibrillation or ventricular tachycardia). To select the candidate predictors for the multivariable models, only those predictors were considered that were present in ≥75% of observations and recorded in the majority of data sets. For the multivariable flexible parametric survival models, backwards selection under Bayesian information criteria stopping rule was applied

The sudden cardiac death risk was low in patients with severely reduced LVEF and very low in mildly reduced or preserved LVEF (*Table 2*). The risk for the primary outcome was considerable in the ICD patients (appropriate defibrillator therapy). Left ventricular ejection fraction was a poor predictor of the primary outcome in all three patient subgroups. Upon external validation, LVEF as sole continuous predictor had a *c*-statistic of 0.50 (95% prediction interval 0.49–0.51) for the risk of first appropriate defibrillator therapy at 36 months in ICD patients, 0.53 (95% prediction interval 0.51–0.54) for the risk of sudden cardiac death in non-ICD patients ≤35%, and 0.56 (95% prediction interval 0.37–0.74) in non-ICD patients >35% (*Figure 1*). Risk stratifying the three study populations into low, medium, and high risk based on LVEF produced very similar cumulative incidence curves, particularly in ICD patients and in the non-ICD patients ≤35%. Separation of the curves was marginally better in non-ICD patients >35% (*Figure 2*). Calibration was similarly poor (see Supplementary data online, *Table S3*).

The consideration of further candidate predictors in addition to LVEF did not improve the predictive performance (*Figures 1* and *2*). In all three study populations, the *c*-statistic of all multivariable models, upon external validation, was very close to 0.5 with very wide prediction intervals indicating large heterogeneity across data sets and demonstrating inability to risk stratify based on these models (*Figures 1* and *2*). The predictive performance was moderate in some databases for the non-ICD patients >35% (*Figure 1*), but with large heterogeneity across data sets. Calibration of the models was reasonable on average (observed:expected ratio close to 1; Supplementary data online, *Table S3*), but again with very large heterogeneity across data sets indicating low generalizability of the models across data sets.

### Phase 2 analysis

Magnetic resonance imaging information was available in 2076 patients for the Phase 2 analysis. *Table 1* presents the baseline information of these patients, and *Table 2* gives the cumulative incidence estimates of the primary outcome. Across these data sets, the combination of the 5 SD method for defining core scar and 3 SD minus 5 SD method for defining greyzone consistently performed best at predicting sudden cardiac death risk (see Supplementary data online, *Tables S4* and *S5*). Thus, these definitions were used in the multivariable modelling of Phase 2. Upon external validation, the predictive performance for the primary outcome at 36 months achieved by the model consisting only of LVEF, age, and sex was almost identical with the predictive performance achieved by the model consisting of LVEF, age, sex, extent of core scar, and extent of greyzone, across all three study populations (*Table 3*), indicating a low additive value of the information derived from cardiac magnetic resonance.

**Table 3:** Meta-analysis results of the predictive performance results for the primary endpoint at 36 months for the three study groups in the Phase 2 analysis

Model	*C*-statistic (95% prediction interval)	Observed:expected ratio (95% prediction interval)
ICD patients		
Model without CMR information: age, sex, and left ventricular ejection fraction as predictors	0.51 (0.45, 0.58)	1.10 (0.94, 1.29)
Model with CMR information: age, sex, left ventricular ejection fraction, core scar, and greyzone as predictors	0.53 (0.46, 0.59)	1.09 (0.93, 1.27)
Non-ICD patients ≤35%		
Model without CMR information: age, sex, and left ventricular ejection fraction as predictors	0.40 (0.31, 0.49)	0.94 (0.76, 1.16)
Model with CMR information: age, sex, left ventricular ejection fraction, core scar, and greyzone as predictors	0.54 (0.44, 0.63)	0.91 (0.74, 1.12)
Non-ICD patients >35%		
Model without CMR information: age, sex, and left ventricular ejection fraction as predictors	0.52 (0.36, 0.67)	0.37 (0.27, 0.50)
Model with CMR information: age, sex, left ventricular ejection fraction, core scar, and greyzone as predictors	0.56 (0.42, 0.71)	0.36 (0.27, 0.49)

All models are flexible parametric survival models. ICD patients, patients with left ventricular ejection fraction ≤ 35% who had received a cardioverter-defibrillator implantation for primary prevention of sudden cardiac death. Non-ICD patients ≤35%, patients who did not carry a cardioverter-defibrillator and had a left ventricular ejection fraction ≤ 35%. Non-ICD patients >35%, patients who did not carry a cardioverter-defibrillator and had a left ventricular ejection fraction > 35%.

CMR, cardiac magnetic resonance.

## Discussion

This pooled cohort analysis of 20 international data sets representing 140 204 patients after myocardial infarction provided two main findings: (i) LVEF had poor predictive performance for the risk of sudden cardiac death among patients with severely reduced LVEF and among those with moderately reduced or preserved LVEF, and (ii) the consideration of a large number and wide spectrum of readily available clinical variables did not improve the predictive performance (*Structured Graphical Abstract*). Thus, more accurate risk stratification of patients among these two subgroups and in particular identification of low-risk individuals with severely reduced LVEF as candidates for omission of defibrillator protection or of high-risk individuals with preserved LVEF as candidates for targeted defibrillator protection was not feasible, neither using LVEF nor using various other candidate predictors.

### Limitations of current practice and inherent limitations in risk prediction for sudden cardiac death

Guidelines recommend LVEF ≤ 35% as criterion for primary prevention defibrillator following myocardial infarction^7,8^ based on historical trial evidence. In the present analysis, LVEF had poor predictive performance for sudden cardiac death with *c*-statistics between 0.50 and 0.56 in all three studied populations corroborating previous reports on limitations of LVEF as sole risk stratification tool.^2,3^ Thus, LVEF did not prove useful in further risk stratifying patients with severely reduced LVEF or patients with moderately reduced or preserved LVEF. Notably, the results of this analysis do not prove that LVEF is in general not useful for risk prediction of sudden cardiac death across the whole spectrum of LVEF.

While the limitations of current practice are evident, there is uncertainty about alternative strategies. The current results contrast with previous studies reporting good discrimination of patients at high risk for sudden cardiac death or for appropriate defibrillator intervention achieved by various models.^6,24–26^ Whether the non-generalizability of results obtained in single or few data sets to a greater number of larger data sets and larger populations provides a plausible explanation is speculative. Previous research efforts were hampered by relatively small patient numbers, neglect of patients with preserved LVEF, and focus on certain predictor categories. To overcome these shortcomings, we analysed a large pooled data set of post-infarction patients with data sets of considerable breadth and depth, including a large patient number with mildly reduced or preserved LVEF, and considered the majority of previously proposed predictors. We applied different analytical methods and consistently used competing risks framework to adjust for risk of death by other causes. Systematic internal–external leave-one-data set-out cross-validation was used to assess generalizable predictive model performance.

Nevertheless, the current analysis did not yield a tool that predicted the individual sudden cardiac death risk with satisfactory accuracy. Whether this result is related to the nature of sudden cardiac death and to inherent limitations of respective analyses rendering prediction not feasible cannot be answered. A key inherent limitation lies on correct adjudication of death cause. Misclassifications of cause of death are indeed frequent,^27^ reducing the performance of models for sudden cardiac death prediction. Furthermore, sudden cardiac death is the result of a complex, highly dynamic interplay of multiple factors that is difficult to capture, especially with single-time assessments.

### Limitations of the present analysis

Some other limitations should be noted. A wide variable spectrum was considered. However, variable availability varied across data sets and may have led to exclusion of variables with potential predictive value. Analysis of electrocardiographic data was limited to data derived from conventional surface electrocardiogram (ECG). Previously reported candidate predictors such as T-wave alternans or baroreflex sensitivity were not analysed due to a paucity of analysable data sets containing information on these parameters. Further, although the analysed subset of patients with available cardiac magnetic resonance imaging constitutes with more than 2100 patients a large pooled data set in this setting, the number of observed events was still relatively small limiting the power of the analysis. Additionally, the higher event rates in patients with magnetic resonance information compared with patients without this information may indicate differences among these populations due to the indication for performing magnetic resonance imaging or the design of the respective cohorts. Notwithstanding these limitations, the results of the Phase 2 analysis, which did not demonstrate a predictive value of cardiac magnetic resonance imaging, are noteworthy particularly considering the costs associated with the examination.

As the aim was to derive a risk stratification scheme that would be easily applicable in everyday clinical practice, invasive risk stratification tools such as programmed ventricular stimulation were not considered. Previous data have demonstrated usefulness of invasive risk stratification, particularly if preceded by non-invasive risk stratification in a two-step approach.^28^ Genetic markers were not considered because up to conduction of the analyses, there was not sufficient evidence for an association between specific genetic markers and risk for sudden cardiac death in patients with coronary artery disease in the stable, post-remodelling phase after infarction.^29^ Nevertheless, a recent report indicates a potential role of genetic information.^30^

A further significant limitation of the analysis is the large heterogeneity of the analysed data sets. This was dictated by the need to combine different data sets in order to achieve the size that is necessary for the study of the rare outcome of sudden cardiac death. These differences in design, data, outcome ascertainment, and follow-up of the analysed cohorts limit the strength of the conclusions.

Recently introduced drugs for heart failure treatment such as sodium–glucose co-transporter 2 inhibitors or angiotensin receptor–neprilysin inhibitors were not available or not yet standard at the time of most cohorts. Therefore, it is unclear whether the results of the presented analysis are well applicable in patients treated with contemporary optimal therapy including these recently introduced agents and contemporary revascularization strategies.

### Rates of defibrillator therapies vs. rates of sudden cardiac death

Overall, the sudden cardiac death risk was low in patients with severely reduced LVEF and very low in moderately reduced or preserved LVEF. Notably, the risk of appropriate defibrillator therapy was considerable in defibrillator carriers. This difference between sudden cardiac death rates in patients with LVEF ≤ 35% not carrying a defibrillator and rates of appropriate defibrillator therapies in defibrillator carriers in our analysis is noteworthy and may have different explanations. It is well known that not all arrhythmias treated by the defibrillator would be fatal if left untreated. An analysis restricted to appropriate defibrillator therapies caused by ventricular fibrillation only could have served as a more accurate surrogate in ICD patients. Such an analysis was not feasible, since information on the arrhythmia triggering the appropriate defibrillator therapy was not consistently available in the analysed data sets. Differences in patient characteristics between these two study populations due to selection bias may have further contributed to the observed differences in endpoint rates. Programming parameters of the devices, which were not controlled for in the analysis, have also an impact on the rates of defibrillator therapy.

### Implications of the findings

These findings have substantial implications in the field of sudden cardiac death prevention after myocardial infarction. In patients with severely reduced LVEF, the lack of appropriate risk stratification tools questions the feasibility of approaches for personalized decision-making on defibrillator implantation. Considering the declining risk for sudden death,^10^ the effect of recently introduced heart failure drugs,^31,32^ the fact that non-sudden deaths account for the large majority of deaths in this population, and the still considerable complication rate of the devices,^33^ a re-evaluation of the benefit of routine prophylactic defibrillator implantation in patients with LVEF ≤ 35% appears necessary.

Similarly, in patients with LVEF > 35%, the lack of acceptably accurate risk stratification tools combined with the very low sudden cardiac death risk questions the feasibility of attempts at identification of high-risk candidates for targeted protection by defibrillator.

## Conclusions

In patients with previous myocardial infarction, LVEF had poor predictive performance for the risk of sudden cardiac death among patients with severely impaired LVEF and among those with moderately reduced or preserved LVEF. The consideration of a large variety and wide spectrum of further candidate predictors did not improve the predictive performance. Thus, more accurate risk stratification and in particular identification of low-risk individuals with severely reduced LVEF as candidates for omission of defibrillator protection or of high-risk individuals with preserved LVEF as candidates for targeted defibrillator protection was not feasible, neither using LVEF nor using various other candidate predictors.

## Supplementary Material

ehae326_Supplementary_Data

## Declarations

### Data Availability

The data that support the findings of this study are available from the corresponding author upon reasonable request. Raw data, including de-identified participant data, statistical code, and supplementary material, will be provided to researchers who meet the criteria for access, following a formal request to the corresponding author. Data sharing is subject to applicable data protection laws and regulations, and the sharing of certain data may require further permissions or ethical approvals. Researchers interested in obtaining the data should contact N.D. at nikolaos.dagres@dhzc-charite.de to initiate the data request process. Additionally, summary data and relevant findings are provided within the manuscript, tables, and supplementary materials, allowing for a comprehensive understanding of the study’s results. We are committed to promoting transparency and facilitating access to data to foster scientific collaboration and further research in the field of cardiology.

### Pre-registered Clinical Trial Number

None supplied.
