# Correction to: Sudden cardiac death after myocardial infarction: individual participant data from pooled cohorts

**DOI:** 10.1093/eurheartj/ehag167

**Published:** 2026-03-12

This is a correction to: Niels Peek, Gerhard Hindricks, Artur Akbarov (et al), Sudden cardiac death after myocardial infarction: individual participant data from pooled cohorts, *European Heart Journal*, Volume 45, Issue 43, 14 November 2024, Pages 4616–4626, https://doi.org/10.1093/eurheartj/ehae326

The name of the 48th co-author should read: “Ivo Roca-Luque”. In section Disclosure of interest, initials “I.R.” should read “I.R.L”.

The errors are outlined only in this correction notice to preserve the version of record.

