# Supplementary material for: Sudden cardiac death after myocardial infarction: individual participant data from pooled cohorts
Source: Eur Heart J. 2024 Oct 8;45(43):4616–26. doi: 10.1093/eurheartj/ehae326 (PMC11560274; doi:10.1093/eurheartj/ehae326)
Supplement: ehae326_Supplementary_Data [file ehae326_supplementary_data.docx]

# Supplementary Material

**Description of datasets**

**Supplementary Table 1**: Common data model showing the consistent set of variables across all data sources. In particular, the common data model dictates the units of measurement where applicable, categories for nominal and ordinal variables, and definitions of each variable.

**Supplementary Table 2**: Pre-selected variables

**Supplementary Table 3**: Meta-analysis results of the predictive performance results for the primary outcome at 12 and 36 months, for the three study groups in phase 1 analysis

**Supplementary Table 4:** Values of myocardial fibrosis and greyzone mass as measured by cardiac magnetic resonance imaging applying different definitions in the phase 2 analysis.

**Supplementary Table 5**: Predictive performance of different definitions of greyzone across datasets as analysed using systematic leave-one-dataset-out cross-validation

**Supplementary Figure 1**: PROFID risk regions on a map of Europe

**Supplementary Figure 2**: PROFID risk regions on the world map

**Supplementary Figure 3**: Fine and Grey cumulative incidence of sudden cardiac death / first appropriate therapy, and death from other causes, for the phase 1 analysis.

**Description of datasets**

1. ***Aston Research Database [ASTN]***

Aston research database is a collection of data from patients with coronary artery disease who underwent cardiac magnetic resonance imaging during the period from 2010 to 2017. None of the patients had implantable cardioverter defibrillator at the time of cardiac magnetic resonance imaging. Some patients received an implantable cardioverter defibrillator during the follow-up period. The first cardiac magnetic imaging date was in July 2010 and the last in August 2017. The last follow-up was in May 2020. The primary endpoint was sudden cardiac death, life-threatening arrhythmia, ventricular tachycardia or ventricular fibrillation (even if it did not lead to sudden cardiac death) and appropriate therapies (in patients with implantable cardioverter defibrillator implants). A sudden cardiac death was defined as a natural, unexpected death due to cardiac causes, heralded by an abrupt loss of consciousness within 1h of the onset of acute symptoms. Sustained ventricular tachycardia was defined as a ventricular rhythm faster than 100 bpm lasting at least 30 seconds. Causes of death were adjudicated on the basis of hospital records and correspondence from primary care physicians. Deaths were classified as unknown if no definitive data was found in hospital or primary care records ^1^.

1. ***Centro Cardiologico Monzino Registry [DRVT]***

Centro Cardiologico Monzino Registry is a prospective observational registry which includes patients with chronic heart failure and reduced left ventricular ejection fraction in 34 sites in Europe. Patients underwent clinical evaluation as cardiac magnetic resonance and transthoracic echocardiography. Data was collected from April 2005 to June 2017. The primary endpoint was sudden cardiac death for patients without implantable cardioverter defibrillator and first appropriate therapy for patients with implantable cardioverter defibrillator. Sudden cardiac death was defined as unexpected death either within 1 hour of cardiac symptom onset in the absence of progressive deterioration, during sleep, or within 24 hours of last being seen alive. Event ascertainment is determined by direct interview during office visits or telephone contact with the patient or the patient's immediate family in case of death. Moreover, patient's primary care physician or cardiologist and review of medical records represent further means of information (ex. implantable cardioverter defibrillator interrogation in patients receiving the device and 24-hour ECG-Holter monitoring) ^2^.

1. ***DO-IT Registry [DOIT]***

The Dutch outcome in implantable cardioverter-defibrillator therapy Registry is a nationwide cohort, which recruited 1,500 primary prevention implantable cardioverter defibrillator patients with reduced left ventricular ejection fraction among 28 implantable cardioverter defibrillator centres in the Netherlands. The registry started in September 2014 and the data collection period was from 2014 to 2016. The primary endpoint was appropriate implantable cardioverter defibrillator therapy for ventricular tachycardia or ventricular fibrillation, whilst the secondary outcome measures were inappropriate implantable cardioverter defibrillator therapy, death of any cause, hospitalisation for implantable cardioverter defibrillator related complications and cardiovascular reasons. A clinical event committee, consisting of independent cardiologists, assessed all implantable cardioverter defibrillator delivered therapies by analysing stored electrograms. The committee also determined whether deaths are of cardiac origin ^3,4^.

1. ***ARTEMIS [ATMS]***

ARTEMIS is a prospective and observational study that included patients with proof of coronary artery disease, with or without type 2 diabetes. The study recruited participants at the Division of Cardiology of Oulu University Hospital from August 2007 to December 2012. The primary end point was sudden cardiac death or resuscitation from sudden cardiac arrest, whichever occurred first. The definition for sudden cardiac death was a witnessed death within 1 hour of the onset of symptoms. For unwitnessed deaths, the definition was last being seen alive and stable 24 hours before discovery. A medicolegal autopsy is mandatory in Finland according to the law, and thus autopsy data were available in most cases. End points were determined from emergency rescue reports, hospital and physician records, autopsy data, death certificates, and interviews with the next of kin. The cause and mode of death were reviewed and adjudicated by 2 independent investigators ^5^.

1. ***DAI-PP pilot registry [FREN]***

Défibrillateur Automatique Implantable-Prévention Primaire pilot registry is a retrospective analysis of subjects with ischaemic or non-ischaemic cardiomyopathy, who received implantable cardioverter defibrillator for primary prevention between 2002 and 2012 in 12 French implantable cardioverter defibrillator implantation centres. Implantable cardioverter defibrillator implantations were restricted to the period from 1 January 2002 to 31 December 2013. First implant was in July 2002 and the last in May 2012. Appropriate implantable cardioverter defibrillator therapy was defined as the successful termination of an episode of sustained ventricular tachycardia or ventricular fibrillation by single or multiple shocks, anti-tachycardia pacing, or both. The date of first appropriate implantable cardioverter defibrillator therapy and the number of appropriate therapies delivered during follow-up were recorded. Implantable cardioverter defibrillator -related, nonfatal, late adverse events included infections, lead dislodgement or dysfunction and inappropriate therapies. Implantable cardioverter defibrillator threshold for therapies included, ventricular tachycardia > 180 bpm; ventricular fibrillation above 220 bpm. The vital status was ascertained by review of the patients’ medical files from the hospital or by communication with primary physicians and finally corroborated with the French vital status database of the National Institute of Economic Statistics. In case of death, the specific cause(s) was collected from the patients’ files, complemented by information from the national database available at the French Centre on Medical Causes of Death (Centre d’Epidémiologie sur les Causes Médicales de Décès–CépiDc, Inserm). Each death was reviewed by two investigators and, whenever possible, classified as sudden arrhythmic or non-arrhythmic, end stage heart failure, implantable cardioverter defibrillator-related, or from another cardiovascular, or non-cardiovascular cause ^6^.

1. ***EU-CERT-ICD Retrospective part [CERT]***

The European Comparative Effectiveness Research to Assess the Use of Primary Prophylactic implantable cardioverter defibrillators is a retrospective compilation of 14 locally existing mostly prospective registries of primary prevention French implantable cardioverter defibrillator implantations between 2002 and 2014. Data was collected from patients who received implantable cardioverter defibrillator implants in the period from February 2002 to December 2014. Selected endpoints were all-cause mortality and appropriate implantable cardioverter defibrillator shock delivery, which was considered as the best surrogate parameter for prevented sudden cardiac death. Adjudication was site-specific from 14 local registries in 11 European countries ^7^.

1. ***Heart Center Leipzig [HELS]***

Heart Center Leipzig is a dataset of patients who suffered myocardial infarction and received implantable cardioverter defibrillator implantation for primary prevention of sudden cardiac death. A subset of patients underwent cardiac magnetic resonance imaging evaluation. Data was collected from 2004 to 2020 and the follow-up start was assumed to be the first received implantable cardioverter defibrillator implantation date. Outcomes were assessed as first appropriate therapy as a proxy for sudden cardiac death.

1. ***ISAR-RISK [ISAR]***

ISAR-RISK was a prospective cohort study that recruited patients who survived from an acute myocardial infarction after 30 days from hospitalisations. The study recruited patients between January 1996 and April 2000. The follow-up start was assumed to be the study enrolment date. The primary endpoint of the study was all-cause mortality within the first 5 years of follow-up; secondary endpoints were cardiac and sudden cardiac death also at 5 years of follow-up. Cardiac death was defined as sudden if it was (1) a witnessed death occurring within 60 min of the onset of new symptoms, unless there was an obvious non-cardiac cause, (2) an unwitnessed death within 24h in the absence of pre-existing progressive circulatory failure or other causes of death, or (3) death during attempted resuscitation ^8^.

1. ***Israeli National ICD Registry [ISRL]***

The Israeli National implantable cardioverter defibrillator registry is a prospective cohort for all patients with implantation or replacement of an implantable cardioverter defibrillator or CRT defibrillator in 21 centres in Israel and implantable cardioverter defibrillator implantations were restricted to the period from 2010 to 2015. The primary endpoint of the study was first appropriate therapy / first appropriate shock for patients with implantable cardioverter defibrillator implants. Mortality information was obtained from the Israeli National Population Register ^9^.

1. ***MADIT II Randomised Trial [MDII]l***

The Multicentre Automatic Defibrillator Implantation Trial II started in July 1997 and recruited patients from 76 hospital centres in the United States and Europe. Data was collected from 1997 to 2001 and the follow-up start was assumed to be the first implantable cardioverter defibrillator implantation date for implantable cardioverter defibrillator patients and the enrolment date for non-implantable cardioverter defibrillator patients. Any participants who had principal investigator’s opinions of cause of implantable cardioverter defibrillator action as ventricular tachycardia or ventricular fibrillation were considered as having appropriate therapy. Sudden cardiac death is defined as any sudden and unexpected death within less than five minutes of acute cardiac symptoms, including, if the heart stops without recently preceding active symptoms and/or signs of cardiac disease or if an autopsy examination is performed and does not show conditions such as myocardial or aneurysmal rupture which could account for the abrupt and instantaneous demise by mechanisms initially resuscitated, but the patient dies without regaining consciousness during the associated hospitalisation, the mechanism of death would still be primary cardiac arrhythmias / sudden cardiac death ^10^.

1. ***MADIT RIT Randomised Trial [MDRT]***

The Multi-centre Automatic Defibrillator Implantation Trial: Reduce Inappropriate therapy (MADIT-RIT) clinical trial was a prospective and randomised clinical study of all patients with ischaemic or non-ischaemic heart disease, in sinus rhythm and met approved guidelines for primary prevention with an implantable cardioverter defibrillator. The trial was conducted between 2009 and 2011 across 98 hospital centres in United States, Canada, Europe, Israel, and Japan. The follow-up start was assumed to be the first implantable cardioverter defibrillator implantation date. Appropriate therapy was defined as an energy therapy delivered (anti-tachycardia pacing or shock) for ventricular tachycardia or for appropriate sensing. Sudden cardiac death was defined as any subject who experienced SCA survived in a post-resuscitative course but never regained consciousness or function and did not leave the hospital or had an unexpected catastrophic cardiac event that lead to death ^11^.

1. ***Nancy Research Database [NANC]***

Nancy Research Database is a collection of data on patients with cardiac magnetic resonance imaging who underwent implantable cardioverter defibrillator implantation. The follow-up start was assumed to be the first implantable cardioverter defibrillator implantation date. Sudden cardiac death was defined as a natural, unexpected death due to cardiac causes, heralded by an abrupt loss of consciousness within 1 h of the onset of acute symptoms. Composite arrhythmic of sudden cardiac death, resuscitated cardiac arrest, sustained ventricular tachycardia or ventricular fibrillation, or appropriate implantable cardioverter defibrillator shock. Sustained ventricular tachycardia was defined as a ventricular rhythm faster than 100 beats/min lasting at least 30s or requiring termination due to hemodynamic instability or by anti-tachycardia pacing or shocks. In patients with devices, electrograms were reviewed to determine arrhythmias and to decide whether delivered shocks were appropriate or inappropriate. Only appropriate shocks were considered as arrhythmic.

1. ***Olomouc Research Database [OLMC]***

Olomouc is a dataset of patients who suffered myocardial infarction or having coronary heart disease with left ventricular ejection fraction of <50% and received implantable cardioverter defibrillator implants for primary prevention. Implantable cardioverter defibrillator implantations were restricted to the period from 2009 to 2019 and the follow-up start was assumed to be the first received implantable cardioverter defibrillator implantation date. The primary endpoint of the study was first appropriate therapy / first appropriate shock for patients with implantable cardioverter defibrillator implants, whilst the secondary end-point was death from any cause. All patients were followed-up in the implanting centres’ out-patient department every 3-6 months to document cardiovascular and non-cardiovascular deaths based on review of the death certificate issued by the attending physician.

1. ***PRE-DETERMINE [PRDT]***

PRE-DETERMINE study and DETERMINE Registry were prospective cohort studies that comprised of patients with coronary disease on angiography or documented history of myocardial infarction. The data was collected from 2007 to 2013 and the follow-up start was assumed to be the study enrolment date. The primary endpoint was a combined endpoint of sudden arrhythmic death. Deaths were classified according to both timing (sudden vs. non-sudden) and mechanism (arrhythmic vs. non-arrhythmic). Unexpected deaths due to cardiac or unknown causes that occurred within 1h of symptom onset or within 24h of being last witnessed to be symptom free were considered sudden cardiac deaths ^12,13^. Deaths preceded by an abrupt spontaneous collapse of circulation without antecedent circulatory or neurologic impairment were considered arrhythmic in accordance with the criteria outlined by Hinkle and Thaler ^14^.

1. ***PROSe-ICD [PRSE] and PROSe LV Structural Predictors Imaging Sub-Study [PSRL]***

PROSE-ICD is a prospective cohort study that recruited patients with primary prevention implantable cardioverter defibrillator or CRT-D in 4 centres in the United States. The study started in December 2003 and the follow-up start was assumed to be the first received implantable cardioverter defibrillator implantation date. The Left Ventricular Structural Predictors of Sudden Cardiac Death registry was a study conducted among 382 patients meeting clinical criteria for primary prevention implantable cardioverter defibrillator with left ventricular ejection fraction ≤35% between 2003 and 2015 at three sites in the United States. The follow-up start was assumed to be the CMR imaging date. The primary endpoint was appropriate implantable cardioverter defibrillator shock for ventricular tachycardia above the programmed rate cut-off (generally 180 bpm) or ventricular fibrillation or definite or suspected sudden arrhythmic death. Review of implantable cardioverter defibrillator interrogations when available were done through medical records, death certificates, autopsy reports, and eyewitness accounts. Hinkle-Thaler criteria were used when implantable cardioverter defibrillator interrogations at time of death were unavailable. All arrhythmic events were adjudicated centrally by 2 clinical cardiac electrophysiologists. A third electrophysiologist reconciled disagreements ^15,16^.

1. ***SCD-HeFT trial [SHFT]***

The Sudden Cardiac Death in Heart Failure Trial is a randomised, multi-centre trial conducted at over 125 North American, Australian and New Zealand sites. Patients were enrolled over 2.5 years after being randomly assigned to amiodarone, matched placebo or an implantable cardioverter defibrillator. Median follow-up was 45.5 months. Implantable cardioverter defibrillator therapy was intentionally selected to consist of shock-only, single-lead therapy. The goal was to treat only rapid, sustained ventricular tachycardia or ventricular fibrillation. Sudden cardiac death was assessed as any patients who had death classified as cardiac, sudden and caused by arrhythmia (Tachy or Brady)” ^17^.

1. ***Silesian Centre Research Database [SLSN]***

Silesian Centre Research Database is a collection of data on patients who experienced acute myocardial infarction I. Data was collected from January 2012 to December 2013. Follow-up start for patients was taken any time after the 40 days of the heart remodelling period except for 22 patients. In these patients the time of hospitalisation for myocardial infarction was taken as the follow-up start. Outcome measures included, all-cause mortality, sudden cardiac death and first appropriate therapy for patients with implantable cardioverter defibrillator implants. Sudden cardiac death was defined as a sudden, unexpected death occurring within one hour of the onset of symptoms in a previously stable individual, without evidence of a non-cardiac cause. The death should be witnessed or occur within 24 hours of the person being observed alive and well. Data on long-term all-cause mortality (including mode of death), were collected from a database of the insurer, covering over 99% of population - the National Fund of Health. In patients implanted with implantable cardioverter defibrillator, occurrence of appropriate implantable cardioverter defibrillator therapy and sudden cardiac death were classified based on reviews of device memory, including EGMs print-outs and hospital / outpatient records drawn up during patient’s visits and device check-ups, and if unavailable – on death certificates, hospital records, outpatient notes, contact with relatives or witnesses ^18,19^.

1. ***Swedish Heart Registry [SWHR]***

Swedish Heart Registry represents a combination of 3 registries: SWEDEHEART registry (Swedish Web-System for Enhancement and Development of Evidence-Based care in Heart Disease Evaluated According to Recommended Therapies) including data from the Register of Information and Knowledge About Swedish Heart Intensive Care Admissions (RIKS–HIA), the Swedish Coronary Angiography and Angioplasty Registry, and the Swedish Heart Surgery Registry; the Swedish cardiopulmonary resuscitation registry; and the Swedish Pacemaker and implantable cardioverter defibrillator registry. Data was collected from 2006 to 2017 and the follow-up start was set to 40 days from acute myocardial infarction hospital admission. The primary outcome was sudden cardiac arrest ^20^.

***20.*** ***Barcelona MRI Registry [BARCA]***

The Barcelona MRI registry is a collection of 224 consecutive patients with ischemic and non-ischemic cardiomyopathy, who underwent CMR between 2011 and 2017 before implantable cardioverter defibrillator implantation for primary prevention. The primary end point was appropriate implantable cardioverter defibrillator therapy (anti-tachycardia pacing or shock) for ventricular tachycardia or ventricular fibrillation. The secondary end point was all-cause mortality. 91 Ischaemic patients are considered for the PROFID study ^21^.

**Supplementary Table 1:** Common data model showing the consistent set of variables across all data sources. In particular, the common data model dictates the units of measurement where applicable, categories for nominal and ordinal variables, and definitions of each variable.

| **Classification** | **Variable name** | **Categories/Units** | **Description** |
| --- | --- | --- | --- |
| Demographics | Age | years | Age |
|  | Sex | Male/Female | Sex |
| Clinical characteristics | BMI | kg/m^2^ | Body mass index |
|  | SBP | mmHg | Systolic blood pressure |
|  | DBP | mmHg | Diastolic blood pressure |
|  | NYHA | I/II/III/IV | New York Heart Association classification |
|  | MI_type | STEMI/NSTEMI | Myocardial infarction (MI) type at index MI |
|  | MI_location_anterior | No/Yes | Location of index MI - anterior |
|  | MI_location_inferior | No/Yes | Location of index MI - inferior |
|  | MI_location_lateral | No/Yes | Location of index MI - lateral |
|  | MI_location_posterior | No/Yes | Location of index MI - posterior |
|  | PCI | No/Yes | Percutaneous coronary intervention (PCI) prior to or at the time of follow-up start |
|  | CABG | No/Yes | Coronary artery bypass graft surgery (CABG) prior to or at the time of follow-up start |
|  | PCI_acute | No/Yes | PCI during index MI |
|  | CABG_acute | No/Yes | CABG during index MI |
|  | Thrombolysis_acute | No/Yes | Thrombolysis during index MI |
|  | Revascularisation_acute | No/Yes | Revascularisation during index MI (PCI/CABG/thrombolysis) |
|  | Diseased_arteries_num | 0 \| 1 \| 2 \| ≥3 | Number of diseased coronary arteries |
|  | Baseline type MI40d | 0 \| 1 | Index date for observation within 40 days after index infarction |
|  | lsSWHR | 0 \| 1 | Patient included in the SWHR dataset |
| Echocardiographic measurements | LVEF | % | Left ventricular ejection fraction (echocardiography) |
|  | LVDD | mm | Left ventricular diastolic diameter |
| Circulating biomarkers | BUN | mmol/L | Blood urea nitrogen |
|  | Cholesterol | mg/dL | Total cholesterol |
|  | CRP | mg/L | C-reactive protein |
|  | eGFR | mL/min/1.73m^2^ | Estimated glomerular filtration rate (CKD-EPI) |
|  | Haemoglobin | g/dL | Haemoglobin |
|  | HbA1c | % | Haemoglobin A1c |
|  | HDL | mg/dL | High-density lipoprotein cholesterol |
|  | IL6 | pg/mL | Serum interleukin-6 |
|  | LDL | mg/dL | Low-density lipoprotein cholesterol |
|  | NTProBNP | pmol/L | N-terminal pro-BNP |
|  | Potassium | mmol/L | Potassium |
|  | Sodium | mmol/L | Sodium |
|  | Triglycerides | mg/dL | Triglycerides |
|  | Troponin_T | ng/l | Troponin T |
|  | TSH | mU/L | Thyroid-stimulating hormone |
| ECG | HR | bpm | Resting heart rate (12-lead ECG) |
|  | PR | ms | PR interval (12-lead ECG) |
|  | QRS | ms | QRS duration (12-lead ECG) |
|  | QTc | ms | Corrected QT interval (Bazett; 12-lead ECG) |
|  | AV_block | No/Yes | Atrioventricular block |
|  | AV_block_II_or_III | No/Yes | AV block second or third degree |
|  | LBBB | No/Yes | Left bundle branch block |
|  | RBBB | No/Yes | Right bundle branch block |
| Holter monitor | HR_average | bpm | Average heart rate |
|  | SDNN | ms | Standard deviation of NN intervals |
| Medical history | MI_history | No/Yes | History of prior MI before index MI |
|  | Time_index_MI_CHD | months | Time since latest MI or diagnosis of CHD |
|  | HF | No/Yes | Heart failure |
|  | Stroke_TIA | No/Yes | Stroke or transient ischaemic attack |
|  | NSVT | No/Yes | History of non-sustained ventricular tachycardia |
|  | AF_atrial_flutter | No/Yes | Atrial fibrillation or atrial flutter |
|  | Cancer | No/Yes | Cancer |
|  | COPD | No/Yes | Chronic obstructive pulmonary disease |
|  | Dementia | No/Yes | Dementia |
|  | Diabetes | No/Yes | Diabetes |
|  | Hypertension | No/Yes | Hypertension |
|  | MR | None or mild/  Moderate/Severe | Mitral regurgitation |
| Family history | FH_CAD | No/Yes | Family history of coronary artery disease |
|  | FH_SCD | No/Yes | Family history of sudden cardiac death |
| Life style | Alcohol | No/Yes | Alcohol consumption |
|  | Smoking | No/Yes | Smoking |
| Year of baseline | Time_zero_Y | N/A | Calendar year of follow-up start |
| WHO Cardiovascular Disease Risk Region | CVDRR | Low risk/ Moderate risk/ High risk/ Very high risk | Cardiovascular disease risk region category according to the World Health Organization |
| Medication | ACE_inhibitor_ARB | No/Yes | Angiotensin converting enzyme (ACE) inhibitor or angiotensin receptor blocker |
|  | ACE_inhibitor | No/Yes | ACE inhibitor |
|  | ARB | No/Yes | Angiotensin receptor blocker |
|  | Aldosterone_antagonist | No/Yes | Aldosterone antagonist |
|  | Anti_anginal | No/Yes | Anti-anginal |
|  | Anti_arrhythmic_III | No/Yes | Anti-arrhythmic class III |
|  | Anti_coagulant | No/Yes | Oral anti-coagulant |
|  | Anti_diabetic | No/Yes | Anti-diabetic: either insulin based or oral medications. |
|  | Anti_diabetic_insulin | No/Yes | Anti-diabetic |
|  | Anti_diabetic_oral | No/Yes | Oral anti-diabetic |
|  | Anti_platelet | No/Yes | Anti-platelet |
|  | Beta_blockers | No/Yes | Beta-blocker |
|  | Calcium_antagonists | No/Yes | Calcium antagonists |
|  | Digitalis_glycosides | No/Yes | Digitalis glycosides |
|  | Diuretics | No/Yes | Diuretic |
|  | Lipid_lowering | No/Yes | Lipid lowering medication |

**Supplementary Table 2:** Pre-selected variables: predictors that were present in ≥75% of observations and recorded in the majority of datasets.

| **Variables (ICD patients)** | **Variables (Non-ICD patients)** |
| --- | --- |
| Time_zero_Y | Time_zero_Y |
| Sex | Sex |
| Age | Age |
| LVEF | Diabetes |
| NYHA class | Anti diabetic insulin |
| Beta blockers | Anti diabetic |
| ACE inhibitor ARB | Anti diabetic oral |
| Anti platelet | LVEF |
| Diuretics | Diuretics |
| Anti arrhythmic III | Beta blockers |
| CVDRR | Lipid lowering |
| Age*LVEF | ACE inhibitor |
|  | ACE inhibitor |
|  | Hypertension |
|  | AF atrial flutter |
|  | eGFR |
|  | Smoking |
|  | CABG |
|  | Anti platelet |
|  | ARB |
|  | BMI |
|  | PCI |
|  | Time index MI CHD |
|  | Digitalis glycosides |
|  | LBBB |
|  | Calcium antagonists |
|  | Anti anginal |
|  | Anti coagulant |
|  | Haemoglobin |
|  | Baseline type MI40d |
|  | IsSWHR |
|  | CVDRR |
|  | Age*LVEF |
|  | Age*eGFR |
|  | LVEF*Baseline type MI40d |
|  | eGFR*Baseline type MI40d |
|  | Baseline type MI40d*Haemoglobin |

For explanation of the brief variable names please see Supplementary Table 1. The asterisks indicate interactions between variables.

**Supplementary Table 3:** Meta-analysis results of the predictive performance results for the primary endpoint at 12 and 36 months, for the three study groups in phase 1 analysis.

| **Population** | **Model** | **O:E Ratio (95% Prediction Interval)** |
| --- | --- | --- |
| **12 months** | | |
| **ICD patients** | Survival model with left ventricular ejection fraction as sole predictor | 0.93 (0.19, 4.44) |
| **ICD patients** | Multivariable flexible parametric survival model | 0.25 (0.04, 1.35) |
| **ICD patients** | Multivariable random forest survival model | 1.01 (0.21, 4.95) |
| **non-ICD patients ≤35%** | Survival model with left ventricular ejection fraction as sole predictor | 2.23 (0.17, 28.75) |
| **non-ICD patients ≤35%** | Multivariable flexible parametric survival model | 1.33 (0.07, 24.28) |
| **non-ICD patients ≤35%** | Multivariable random forest survival model | 1.38 (0.17, 11.39) |
| **non-ICD patients >35%** | Survival model with left ventricular ejection fraction as sole predictor | 1.05 (0.23, 4.73) |
| **non-ICD patients >35%** | Multivariable flexible parametric survival model | 0.51 (0.03, 10.36) |
| **non-ICD patients >35%** | Multivariable random forest survival model | 0.62 (0.09, 4.18) |
| 36 months | | |
| **ICD patients** | Survival model with left ventricular ejection fraction as sole predictor | 0.88 (0.21, 3.76) |
| **ICD patients** | Multivariable flexible parametric survival model | 0.32 (0.07, 1.37) |
| **ICD patients** | Multivariable random forest survival model | 1.04 (0.24, 4.56) |
| **non-ICD patients ≤35%** | Survival model with left ventricular ejection fraction as sole predictor | 2.02 (0.21, 19.13) |
| **non-ICD patients ≤35%** | Multivariable flexible parametric survival model | 1.31 (0.11, 16.23) |
| **non-ICD patients ≤35%** | Multivariable random forest survival model | 1.47 (0.25, 8.68) |
| **non-ICD patients >35%** | Survival model with left ventricular ejection fraction as sole predictor | 1.24 (0.37, 4.18) |
| **non-ICD patients >35%** | Multivariable flexible parametric survival model | 0.65 (0.05, 8.69) |
| **non-ICD patients >35%** | Multivariable random forest survival model | 0.77 (0.19, 3.18) |

ICD patients: patients with left ventricular ejection fraction ≤35% who had received a cardioverter-defibrillator implantation for primary prevention of sudden cardiac death

non-ICD patients ≤35%: patients who did not carry a cardioverter-defibrillator and had a left ventricular ejection fraction ≤35%

non-ICD patients >35%: patients who did not carry a cardioverter-defibrillator and had a left ventricular ejection fraction >35%.

In ICD patients, endpoint was first appropriate therapy, in non-ICD patients ≤35% and non-ICD patients >35% endpoint was sudden cardiac death. In two datasets with non-ICD patients, the primary endpoint included additionally life-threatening ventricular arrhythmias (ventricular fibrillation or ventricular tachycardia).

O:E = observed divided by expected risk ratio

**Supplementary Table 4:** Values of myocardial fibrosis and greyzone mass as measured by cardiac magnetic resonance imaging applying different definitions in the phase 2 analysis.

| **Variable** | **ICD patients**  **(n = 514)** | **non-ICD patients ≤35%**  **(n = 576)** | **non-ICD patients >35%**  **(n = 986)** |
| --- | --- | --- | --- |
| Total fibrosis _2SD_ (g) | 46.4 (25.5 - 77.8) | 56.9 (30.5 - 82.3) | 37.9 (11.8 - 60.9) |
| Total fibrosis _3SD_ (g) | 37.4 (20.0 - 63.1) | 46.4 (24.3 - 68.7) | 30.5 (8.4 - 50.3) |
| Total fibrosis _5SD_ (g) | 24.5 (9.3 - 44.8) | 30.5 (11.6 - 49.8) | 18.6 (3.2 - 35.4) |
| Total fibrosis _FWHM_ (g) | 25.7 (16.2 - 37.2) | 25.8 (16.9 - 37.5) | 14.8 (7.1 - 24.3) |
| Greyzone mass _2SD-3SD_ (g) | 9.2 (3.2 - 14.9) | 9.6 (5.3 - 15.2) | 6.3 (3.2 - 10.3) |
| Greyzone mass _2SD-5SD_ (g) | 22.1 (7.4 - 35.7) | 25.2 (14.8 - 36.2) | 16.7 (7.4 - 25.3) |
| Greyzone mass _3SD-5SD_ (g) | 12.7 (4.9 - 20.7) | 15.0 (9.4 - 21.1) | 9.8 (4.2 - 14.8) |
| Greyzone mass _2SD-FWHM_ (g) | 14.4 (4.1 - 46) | 28.0 (5.6 - 51.3) | 13.0 (1.1 - 39.7) |

Numbers give median values with interquartile ranges in parentheses.

Subscripts refer to the methods for measurement of total fibrosis and for calculation of greyzone mass^22^: 2SD: 2 standard deviations; 3SD: 3 standard deviations; 5SD: 5 standard deviations; FWHM: full-width-half-maximum.

ICD patients: patients with left ventricular ejection fraction ≤35% who had received a cardioverter-defibrillator implantation for primary prevention of sudden cardiac death

non-ICD patients ≤35%: patients who did not carry a cardioverter-defibrillator and had a left ventricular ejection fraction ≤35%

non-ICD patients >35%: patients who did not carry a cardioverter-defibrillator and had a left ventricular ejection fraction >35%.

**Supplementary Table 5:** Predictive performance of different definitions of core scar and greyzone, across datasets as analysed using systematic leave-one-dataset-out cross-validation

| **Dataset** | **Core scar definition** | **Grey Zone definition** | **C-statistic (95% confidence intervals)** |
| --- | --- | --- | --- |
| ASTN | 3SD | 2SD-3SD | 0.75 (0.71 - 0.79) |
| ASTN | 5SD | 2SD-5SD | 0.76 (0.72 - 0.80) |
| ASTN | 5SD | 3SD-5SD | 0.77 (0.73 - 0.81) |
| ASTN | FWHM | 2SD-FWHM | 0.75 (0.71 - 0.80) |
| BARCA | 3SD | 2SD-3SD | 0.71 (0.66 - 0.75) |
| BARCA | 5SD | 2SD-5SD | 0.72 (0.67 - 0.76) |
| BARCA | 5SD | 3SD-5SD | 0.73 (0.69 - 0.77) |
| BARCA | FWHM | 2SD-FWHM | 0.72 (0.68 - 0.76) |
| DRVT | 3SD | 2SD-3SD | 0.71 (0.66 - 0.76) |
| DRVT | 5SD | 2SD-5SD | 0.72 (0.67 - 0.77) |
| DRVT | 5SD | 3SD-5SD | 0.72 (0.68 - 0.77) |
| DRVT | FWHM | 2SD-FWHM | 0.72 (0.67 - 0.76) |
| HELS | 3SD | 2SD-3SD | 0.71 (0.67 - 0.76) |
| HELS | 5SD | 2SD-5SD | 0.71 (0.67 - 0.75) |
| HELS | 5SD | 3SD-5SD | 0.73 (0.69 - 0.77) |
| HELS | FWHM | 2SD-FWHM | 0.72 (0.67 - 0.76) |
| NANC | 3SD | 2SD-3SD | 0.72 (0.67 - 0.77) |
| NANC | 5SD | 2SD-5SD | 0.72 (0.68 - 0.77) |
| NANC | 5SD | 3SD-5SD | 0.74 (0.69 - 0.78) |
| NANC | FWHM | 2SD-FWHM | 0.71 (0.66 - 0.75) |
| PRDT | 3SD | 2SD-3SD | 0.69 (0.65 - 0.73) |
| PRDT | 5SD | 2SD-5SD | 0.69 (0.65 - 0.73) |
| PRDT | 5SD | 3SD-5SD | 0.70 (0.66 - 0.74) |
| PRDT | FWHM | 2SD-FWHM | 0.70 (0.66 - 0.74) |
| PRSE | 3SD | 2SD-3SD | 0.72 (0.67 - 0.76) |
| PRSE | 5SD | 2SD-5SD | 0.71 (0.66 - 0.76) |
| PRSE | 5SD | 3SD-5SD | 0.72 (0.67 - 0.77) |
| PRSE | FWHM | 2SD-FWHM | 0.71 (0.66 - 0.75) |

FWHM: full-width-half-maximum, 2SD: 2 standard deviations method, 3SD: 3 standard deviations method and 5SD: 5 standard deviations method^22^.

For abbreviations of datasets please see the Description of datasets in the Supplementary Material.

**Supplementary Figure 1**: PROFID risk regions on a map of Europe.


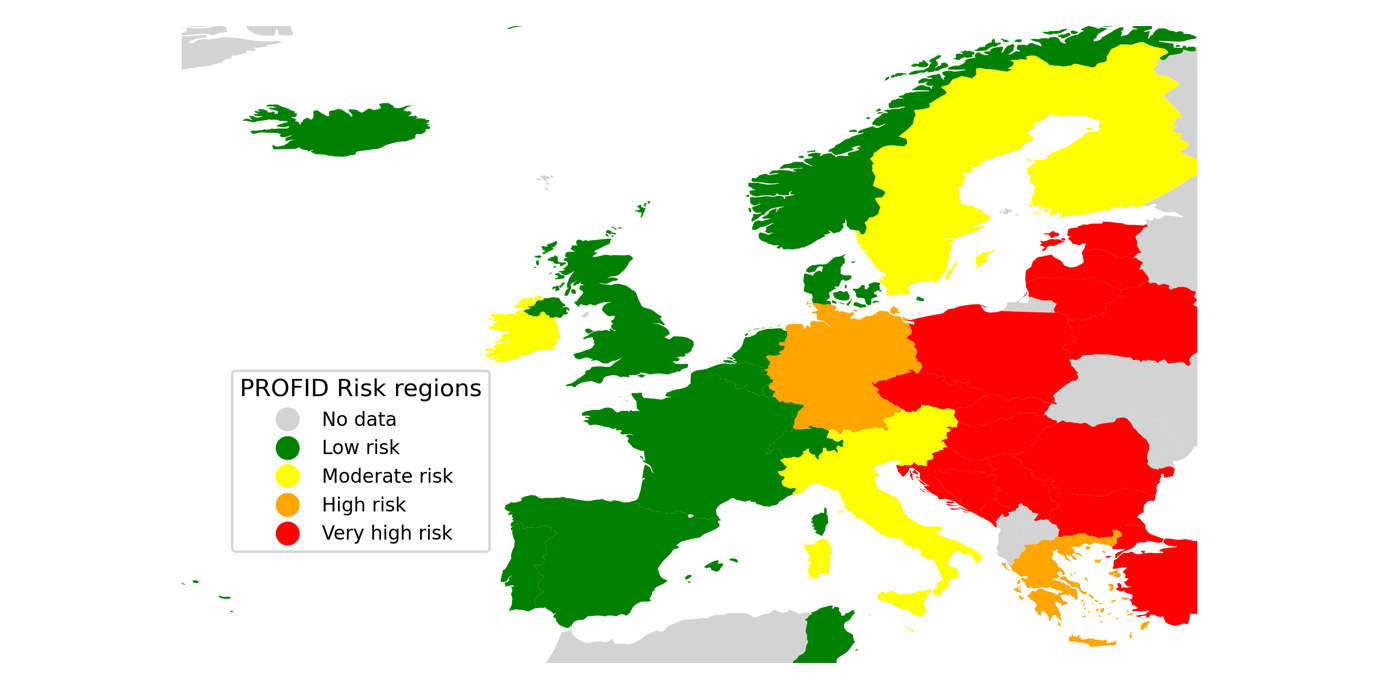


Legend:

Following the previously described approach used for the SCORE2 risk prediction algorithms^23^, a categorical variable was created to capture geographical variations in baseline cardiovascular risk, and this variable was included in all our analyses. We identified four risk regions by thresholding the age-standardised rates of cardiovascular mortality in the countries that contributed data to our study. Each patient in the datasets was then assigned to the corresponding risk region, based on their country of origin. Defining the risk regions was a three-step approach. First, World Health Organisation (WHO) mortality and population data were cross-referenced to compute the yearly age-specific CVD mortality; then, the obtained mortality was projected onto the WHO 2000-2025 standard population (https://www.who.int/data/data-collection-tools/who-mortality-database); finally, the PROFID countries were partitioned into four subsets based on their WHO-standardised CVD mortality.

**Supplementary Figure 2**: PROFID risk regions on the world map.


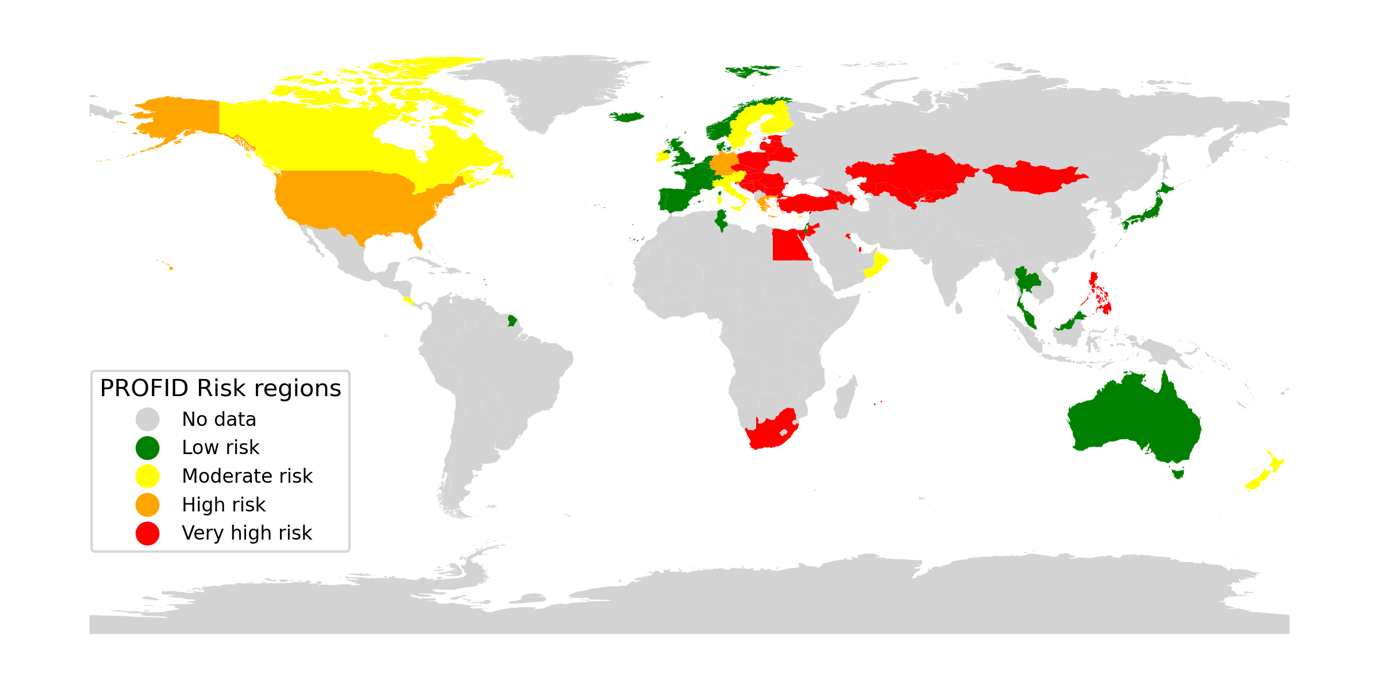


Legend:

For methodology, please see Supplementary Figure 1.

**Supplementary Figure 3:** Fine and Grey cumulative incidence of the endpoint (red), and death from other causes (cyan), for the phase 1 analysis.

A = ICD Patients; B = Non-ICD patients ≤35%; C = Non-ICD patients >35%.

**
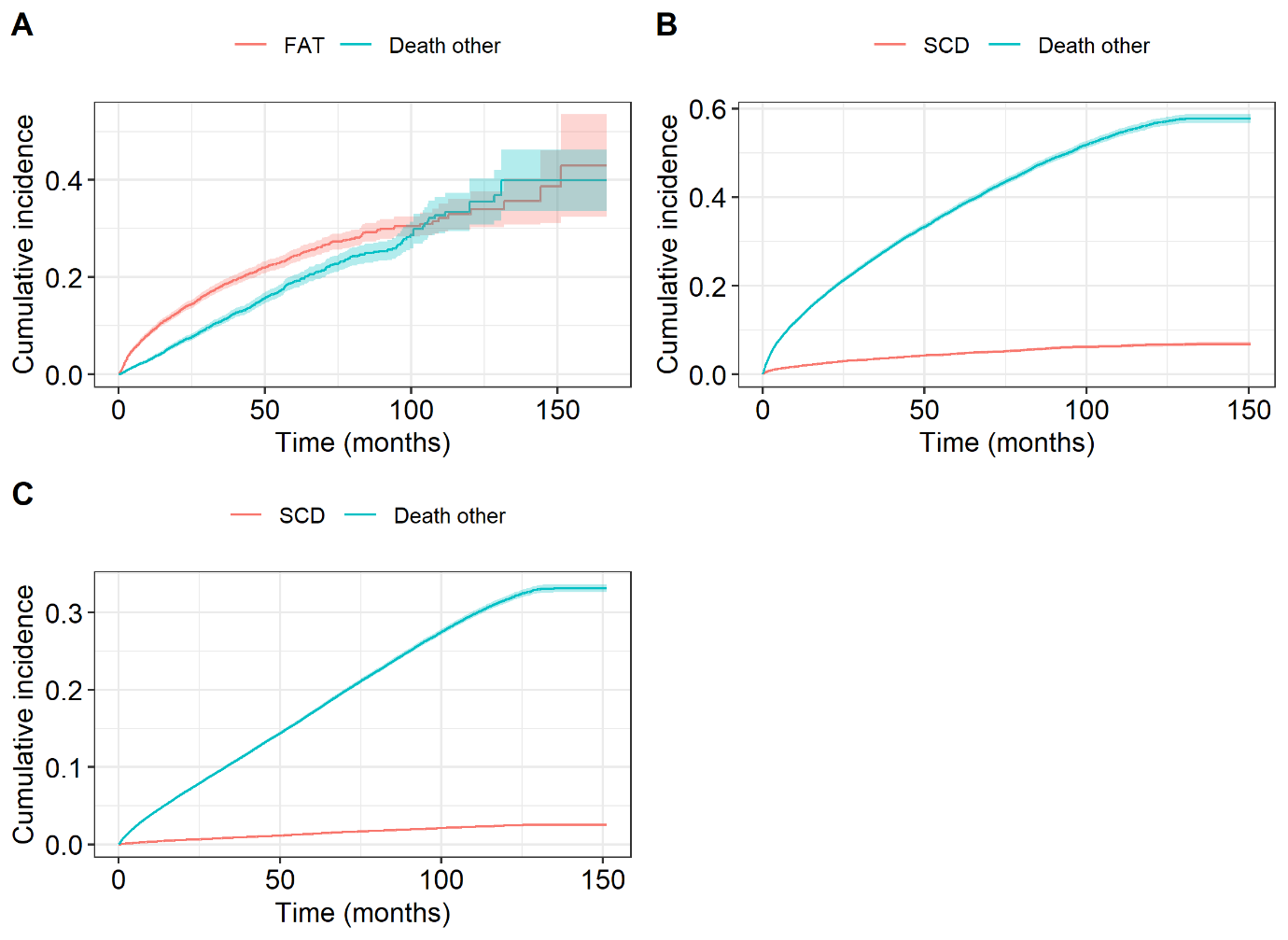
**

Legend:

ICD patients: patients with left ventricular ejection fraction ≤35% who had received a cardioverter-defibrillator implantation for primary prevention of sudden cardiac death

non-ICD patients ≤35%: patients who did not carry a cardioverter-defibrillator and had a left ventricular ejection fraction ≤35%

non-ICD patients >35%: patients who did not carry a cardioverter-defibrillator and had a left ventricular ejection fraction >35%.

In ICD patients, endpoint was first appropriate therapy (FAT), in non-ICD patients ≤35% and non-ICD patients >35% endpoint was sudden cardiac death (SCD). In two datasets with non-ICD patients, the primary endpoint included additionally life-threatening ventricular arrhythmias (ventricular fibrillation or ventricular tachycardia).

During follow-up, a total of 2,227 deaths from any cause occurred in ICD patients, 10,877 in Non-ICD patients ≤35%, and 21,489 in Non-ICD patients >35%.

**References of the supplementary material**

1. Zegard A, Okafor O, Bono J de, Kalla M, Lencioni M, Marshall H, et al. Myocardial Fibrosis as a Predictor of Sudden Death in Patients With Coronary Artery Disease. *Journal of the American College of Cardiology* 2021;**77**:29–41.

2. Guaricci AI, Masci PG, Lorenzoni V, Schwitter J, Pontone G. CarDiac MagnEtic Resonance for Primary Prevention Implantable CardioVerter DebrillAtor ThErapy international registry: Design and rationale of the DERIVATE study. *International Journal of Cardiology* **261**:223–227.

3. Barreveld M Van, Dijkgraaf MGW, Hulleman M, Boersma LVA, Delnoy PPHM, Meine M, et al. Dutch outcome in implantable cardioverter-defibrillator therapy (DO-IT): Registry design and baseline characteristics of a prospective observational cohort study to predict appropriate indication for implantable cardioverter-defibrillator. *Netherlands Heart Journal* 2017;**25**:574–580.

4. Verstraelen TE, Barreveld M van, Dessel PHFM van, Boersma LVA, Delnoy P-PPHM, Tuinenburg AE, et al. Development and external validation of prediction models to predict implantable cardioverter-defibrillator efficacy in primary prevention of sudden cardiac death. *Europace* 2021;**23**:887–897.

5. Junttila MJ, Kiviniemi AM, Lepojärvi ES, Tulppo M, Piira O-P, Kenttä T, et al. Type 2 diabetes and coronary artery disease: Preserved ejection fraction and sudden cardiac death. *Heart Rhythm* 2018;**15**:1450–1456.

6. Boveda S, Narayanan K, Jacob S, Providencia R, Algalarrondo V, Bouzeman A, et al. Temporal Trends Over a Decade of Defibrillator Therapy for Primary Prevention in Community Practice. *Journal of Cardiovascular Electrophysiology* 2017;**28**:666–673.

7. Sticherling C, Arendacka B, Svendsen JH, Wijers S, Friede T, Stockinger J, et al. Sex differences in outcomes of primary prevention implantable cardioverter-defibrillator therapy: combined registry data from eleven European countries. *Europace* 2018;**20**:963–970.

8. Ubrich R, Barthel P, Haller B, Hnatkova K, Huster KM, Steger A, et al. Sex differences in long-term mortality among acute myocardial infarction patients: Results from the ISAR-RISK and ART studies. *PLoS ONE* 2017;**12**:1–17.

9. Sabbag A, Suleiman M, Laish-Farkash A, Samania N, Kazatsker M, Goldenberg I, et al. Contemporary rates of appropriate shock therapy in patients who receive implantable device therapy in a real-world setting: From the Israeli ICD Registry. *Heart Rhythm* 2015;**12**:2426–2433.

10. Moss AJ, Zareba W, Hall WJ, Klein H, Wilber DJ, Cannom DS, et al. Prophylactic Implantation of a Defibrillator in Patients with Myocardial Infarction and Reduced Ejection Fraction. *New England Journal of Medicine* 2002;**346**:877–883.

11. Moss AJ, Schuger C, Beck CA, Brown MW, Cannom DS, Daubert JP, et al. Reduction in Inappropriate Therapy and Mortality through ICD Programming. *New England Journal of Medicine* 2012;**367**:2275–2283.

12. Chatterjee NA, Tikkanen JT, Panicker GK, Narula D, Lee DC, Kentta T, et al. Simple electrocardiographic measures improve sudden arrhythmic death prediction in coronary disease

13. Chatterjee NA, Levy WC. Sudden cardiac death after myocardial infarction. *European Journal of Heart Failure* 2020:5–7.

14. Hinkle LEJ, Thaler HT. Clinical classification of cardiac deaths. *Circulation* 1982;**65**:457–464.

15. Cheng A, Dalal D, Butcher B, Norgard S, Zhang Y, Dickfeld T, et al. Prospective observational study of implantable cardioverter-defibrillators in primary prevention of sudden cardiac death: study design and cohort description. *Journal of the American Heart Association* 2013;**2**.

16. Wu KC, Wongvibulsin S, Tao S, Ashikaga H, Stillabower M, Dickfeld TM, et al. Baseline and Dynamic Risk Predictors of Appropriate Implantable Cardioverter Defibrillator Therapy. *Journal of the American Heart Association* 2020;**9**:e017002.

17. Bardy GH, Lee KL, Mark DB, Poole JE, Packer DL, Boineau R, et al. Amiodarone or an implantable cardioverter-defibrillator for congestive heart failure. *The New England journal of medicine* 2005;**352**:225–237.

18. Francuz P, Podolecki T, Kozieł M, Kalarus Z, Kowalczyk J. Major adverse cardiovascular events in patients after acute myocardial infarction treated invasively and different patterns of glucometabolic disturbances evaluated at mid-term follow-up. *Journal of Medical Science* 2017;**86**:17–22.

19. Francuz P, Podolecki T, Przybylska-Siedlecka K, Kalarus Z, Kowalczyk J. Long-term prognosis is related to mid-term changes of glucometabolic status in patients with acute myocardial infarction treated invasively. *Kardiol Pol* 2017;**75**:117–125.

20. Faxén J, Jernberg T, Hollenberg J, Gadler F, Herlitz J, Szummer K. Incidence and Predictors of Out-of-Hospital Cardiac Arrest Within 90 Days After Myocardial Infarction. *Journal of the American College of Cardiology* 2020;**76**:2926–2936.

21. Sánchez-Somonte P, Quinto L, Garre P, Zaraket F, Alarcón F, Borràs R, et al. Scar channels in cardiac magnetic resonance to predict appropriate therapies in primary prevention. *Heart Rhythm* 2021;**18**:1336–1343.

22. Schulz-Menger J, Bluemke DA, Bremerich J, Flamm SD, Fogel MA, Friedrich MG, et al. Standardized image interpretation and post-processing in cardiovascular magnetic resonance - 2020 update : Society for Cardiovascular Magnetic Resonance (SCMR): Board of Trustees Task Force on Standardized Post-Processing. *J Cardiovasc Magn Reson* 2020;**22**:19.

23. SCORE2 risk prediction algorithms: new models to estimate 10-year risk of cardiovascular disease in Europe. *Eur Heart J* 2021;**42**:2439–2454.
